# Incidence and risk factors of herpes zoster among hiv-positive patients in the german competence network for HIV/AIDS (KompNet): a cohort study analysis

**DOI:** 10.1186/1471-2334-13-372

**Published:** 2013-08-10

**Authors:** Klaus Jansen, Burkhard Haastert, Claudia Michalik, Adrienne Guignard, Stefan Esser, Stephan Dupke, Andreas Plettenberg, Adriane Skaletz-Rorowski, Norbert H Brockmeyer

**Affiliations:** 1 Competence Network for HIV/AIDS, Ruhr-University, Gudrunstrasse 56, 44791 Bochum, Germany; 2 Department of Dermatology, Venerology, and Allergology, St. Josef-Hospital, Ruhr-Universität Bochum, Gudrunstrasse 56, 44791 Bochum, Germany; 3 MediStatistica, Lambertusweg 1b, 58809 Neuenrade, Germany; 4 Centre for Clinical Trials, Gleueler Strasse 269, 50935 Cologne, Germany; 5 GSK Biologicals, Parc de la Noire Epine, Rue Fleming, 20, 1300 Wavre, Belgium; 6 Clinic of Dermatology, University Clinic, Hufelandstrasse 55, 45147 Essen, Germany; 7 Private practice Driesener Strasse, Driesener Strasse 11, 10439 Berlin, Germany; 8 Ifi-Institute, Asklepios-Clinic, Lohmühlenstrasse 5, 20099 Hamburg, Germany

**Keywords:** Herpes zoster, HIV, Highly active antiretroviral therapy, Risk factor analysis

## Abstract

**Background:**

HIV infection is a risk factor for the development of Herpes zoster (HZ) and its complications. Prior to antiretroviral therapy (ART), HZ incidence in HIV-infected individuals ranged from 2.9–5.1/100 person-years. There is limited evidence for the impact of ART on HZ occurrence among HIV-infected adults. We analysed the incidence of, and risk factors for, HZ in a large cohort of German HIV-positive patients.

**Methods:**

The study population was taken from the German KompNet cohort, a nationwide multicenter HIV cohort study. The study population was defined by age (≥ 18 years), year of first positive HIV diagnosis, CD4 values ± 6 months from HIV diagnosis (t_0_), and month of HZ diagnosis. Incidences were estimated using a Poisson distribution, and uni- and multivariate Cox proportional Hazard ratio (HR) regression models were fitted to identify risk factors for developing an initial HZ episode. Independent variables were sex, age at HIV diagnosis, route of HIV transmission, ART status, CD4 count before HZ episode, immunosuppressive medication, and mode of data documentation (retrospective or prospective).

**Results:**

HZ incidence in the overall study population was 1.2/100 person-years. In a subset of patients for that we were able to examine risk factors the following was observed: We examined 3,757 individuals whose mean age at t_0_ was 38 years. Of those individuals, 96% were diagnosed with HIV in 1996 or later, with a mean observation time of 5.8 years. HZ episodes (*n* = 362) were recorded in 326 patients (8.7%), resulting in annual HZ incidences of 1.7/100 person-years overall, and 1.6/100 person-years for initial HZ cases. The main risk factors associated with an initial HZ episode were: not partaking in ART compared with an ART regimen containing a non-nucleoside reverse-transcriptase inhibitor (HR 0.530, *p* < 0.001) or a protease inhibitor (HR 0.624, *p* = 0.004); and lower CD4 count by 100 cells/μl (HR 0.918, *p*=0.001).

**Conclusions:**

HZ incidence was 4-11-fold higher than in non HIV-infected individuals, but in our study HZ incidences were lower than in previous studies relating to HIV-positive patients. We showed that ART is an important protective factor for HZ episodes.

## Background

Herpes zoster (HZ) is the clinical manifestation resulting from the reactivation of the varicella zoster virus (VZV), and can be a debilitating illness. The incidence of HZ in the general population is around 0.15–0.33/100 person-years [1-4], with a higher incidence (0.5–0.9/100 person-years) in individuals aged 50–80 years [5]. Incidence increases with age, along with certain conditions that impair cell-mediated immunity. HZ can be severe, resulting in hospitalisation; in some cases serious complications can occur. Human immunodeficiency virus (HIV) infection is a risk factor for the development of HZ and its complications [6]. In cohort studies conducted before the introduction of highly active anti-retroviral therapy (ART), the annual incidence of HZ in HIV-infected people ranged from 2.9–5.1/100 person-years [7-11]. The overall age-adjusted risk of HZ is at least 15-fold higher in homosexual men infected with HIV than in uninfected men without HIV [9]. The advent of ART has seen mortality in HIV-infected patients dramatically decline, and has reduced the incidence of the most common opportunistic infections. However, ART appears to have little effect on the incidence of HZ [12,13] in most groups other than children [14-19]. Results from a recent study suggested a general protective effect of modern ART regimens [20]. There is limited evidence for the impact of ART on the occurrence of HZ among HIV-infected adults. In this study, we analysed the incidence of, and risk factors for, HZ among HIV-infected patients within the German Competence Network for HIV/AIDS (KompNet). KompNet is an open, retrospective and prospective, multicentre disease-specific and nationwide cohort study funded by the German Federal Ministry of Education and Research [21,22].

## Methods

### Study population and data collection

The study population was selected from HIV-infected patients within the KompNet cohort. The cohort study was approved by the relevant ethical committees for all participating centres and was compliant with the Helsinki declaration. Between June 2004 and September 2007, the KompNet cohort comprised 44 documenting sites (21 outpatient clinics and 23 private practitioners) and included 15,381 subjects with HIV/AIDS. In September 2007, it was reduced to 25 documenting sites (10 outpatient clinics, 15 private practitioners) and 8,162 subjects. Most patients were recruited between autumn 2005 and autumn 2006. Cohort participants had approximately two visits annually. During these visits multiple clinical, laboratory, and sociodemographic variables were collected prospectively [21]. The interval length between visits ranged 4–8 months; serum samples were collected at all visits, however, cells were collected for storage at enrolment and 3 years after enrolment. Data was collected prospectively (i.e. at enrollment into the cohort), in addition chart reviews were performed and data was collected retrospectively when available on patients who attend the KompNet clinical sites before enrolment. The starting point for retrospective data collection was the time of HIV diagnosis (referred to as t_0_).

**Figure 1:**
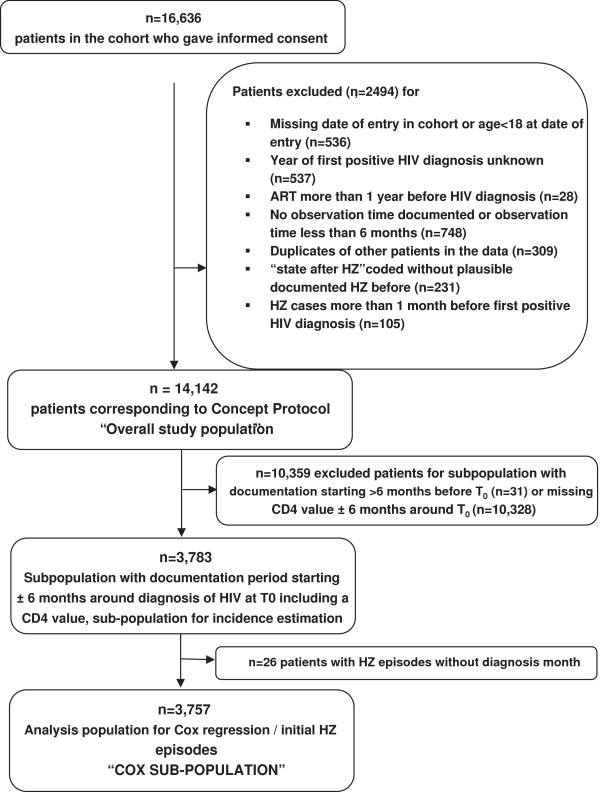
Selection of patients for overall study population and Cox subpopulation.

The following inclusion and exclusion criteria were applied to define the overall study population. Inclusion criteria for subjects required them to be HIV-positive subjects enrolled in the KompNet cohort, over 18-years-old, and provided informed consent for collection and analyses of clinical and socio-demographic data and blood samples. Exclusion criteria were (Figure 1): missing date of enrolment in the cohort; missing year of first plausible positive HIV diagnosis; an interval less than 6-months between the first and last visit during the study period; ART for more than one year before HIV diagnosis; duplicate subjects; documentation of “state after HZ” but HZ never previously documented; HZ more than 1 month before a first positive HIV diagnosis. If the month of diagnosis was not documented, January was considered the month of diagnosis. Additional exclusion criteria were applied to define a sub-population for the analysis of partly time-dependent risk factors associated with HZ (Cox-subpopulation): no documented CD4 count within a 6-month period before or after t_0_; first documentation for either CD4 count or viral load more than 6 months after t_0_; and month of initial HZ diagnosis not available.

### Study period

The analyses described in this report contain data collected from 1 January 1985 until 1 July 2010.

### Identification of HZ cases

In the cohort database, the incidence of HZ cases were coded as monosegmental, multisegmental, multisegmental recurring HZ, or a current HZ episode without other specifications. Cases of HZ were mostly diagnosed on clinical grounds. Additional available documentation of a “condition after Herpes zoster” was used only for validating initial episodes of HZ. For all patients diagnosed with HZ we checked if there was a prior documented diagnosis of “condition after Herpes zoster” or a prior diagnosis of HZ without a documented month. If either were true, patients were excluded from all analyses on initial HZ cases.

HZ cases associated with Immune Reconstitution Inflammatory Syndrome (IRIS) following the initiation of ART were investigated. HZ cases were considered to be associated with IRIS if HZ was diagnosed within 3 months following commencement of ART in ART-naïve patients. If ART-naïvety could not be ascertained, a corresponding variable was imputed as missing value.

### Data analyses

Baseline characteristics were described by frequencies and percentages or by means, standard deviations and ranges, depending on their distributions. Baseline characteristics were compared between the Cox subpopulation and all other patients of the total study population using the χ^2^- and t-tests. The incidence of HZ was calculated for the whole cohort at risk during the study period, based on both historical and prospectively collected data. Single and multiple diagnoses per patients were considered, with incidences of all HZ cases estimated as events by person-years under risk from HIV diagnosis to last date of follow-up or death. Incidences and 95% confidence intervals (CIs) were estimated assuming a Poisson distribution. HZ incidences in the Cox subpopulation, and the overall study population, were compared by fitting a Poisson regression model with a group indicator as the independent variable. Incidences were stratified by age (10-year age groups calculated by calendar year). Cumulative probabilities of initial HZ episodes depending on time since HIV diagnosis were estimated by the Kaplan-Meier method for the Cox-subpopulation.

To determine specific risk factors for time dependent analyses with respect to an initial HZ episode from the time of HIV diagnosis, Cox proportional hazard regression models were fitted [23]. For subjects without any record of a HZ episode, the last observation was taken as censored time of the patient (including death). To avoid bias for the few patients with extremely long observation times, the time under risk was truncated at a fixed maximum value (17.5 years) where at least 10 patients of the cohort were still at risk. Independent variables included baseline characteristics (sex, age at HIV diagnosis, probable route of infection) and time-dependent variables (ART status, CD4 count before the HZ episode, CD4 nadir, adjuvant medications, and mode of documentation).

CD4 values between two follow-up visits, ART status and immunosuppressive medication were assigned using the last observation carried forward (LOCF) principle. All independent variables (continuous or categorised) were first assessed in univariate models. Significant risk factors (*p* < 0.05), or those considered medically important, were then combined in a multivariate model. Some unreasonable variable combinations were excluded from multivariate models; time on ART, and different ART regimen types were not combined in one model, as periods on different ART regimen would then need to be separated. Additional bivariate models were fitted to adjust for univariate models with retrospective documentation. Gender and age were included as possible confounders in the final model to calculate age- and sex-adjusted results. Results were considered statistically significant when *p*-values were less than 0.05, and all test and confidence intervals were calculated as two-sided unless stated otherwise. Statistical analyses were performed using the statistical software package SAS 9.3 (SAS Institute Inc. Cary, NC, USA).

## Results

The overall study population comprised 14,142 patients (Figure 1), with 84.9% of participants male. The Cox sub-population comprised 3,757 subjects for which all respective inclusion criteria were fulfilled. Table 1 summarises the baseline characteristics, showing that these significantly differed between both populations. Effect sizes were small for age, gender, and for the proportion of men who had sex with men (MSM). Subjects were predominantly of Western European origin and of Caucasian ethnicity (91.6%), and nearly two-thirds were MSM. For the overall population, the mean age at HIV diagnosis was 35.1 years, with around two-thirds of all patients diagnosed with HIV diagnosed in 1996 or later. This proportion was higher (96.0%) in the Cox subpopulation. For the overall population, the mean observation time between HIV diagnosis and last documented clinical data was 10.1 years, and the mean duration between HIV diagnosis and inclusion into the cohort was 7.5 years. For the Cox sub-population, the mean observation time was 5.8 years while the mean duration from diagnosis to inclusion in the cohort was 2.8 years. At HIV diagnosis, infectious diseases (excluding HZ) accounted for the highest estimated prevalence of comorbidities (12.0% in the overall population, 23.2% in the Cox sub-population). Use of immune suppressive medication at HIV diagnosis was infrequent, with an estimated prevalence of 0.35% (0.99% in the subpopulation).

**Table 1:** **Baseline characteristics of patients in the overall cohort population and in the Cox**-**subpopulation**

	**Total population(n=14,142)**	**Cox**-**subpopulation(n=3,757)**	**p**-**value**^**1**^
Mean age at HIV diagnosis (t_0_)	35.1 years (SD: 10.2) (range 0.0-77.3)	37.8 years (SD: 10.2) (range 14.1-77.3)	p<0.001
Proportion of men	84.9% (17 missing)	85.9% (2 missing)	p=0.034
Proportion of MSM	63.5% (101 missing)	66.4% (28 missing)	p<0.001
Proportion of IV drug users	6.6% (101 missing)	3.0% (28 missing)	p<0.001
Mean duration between HIV diagnosis and inclusion in the cohort	7.5 years (SD: 6.0)	2.8 years (SD 3.2)	p<0.001
Mean duration between HIV diagnosis and last clinical data	10.1 years (SD: 6.3)	5.8 years (SD: 3.9)	p<0.001
Proportion of timepoint of HIV-diagnosis ≥ 1996	67.3%	96.0%	p<0.001
Infectious comorbidities at HIV diagnosis (excluding HZ)	12.0%	23.2%	p<0.001

1) t- or χ^2^-tests comparing Cox subpopulation (n=3,757) with all other patients of the overall study population (n=10,385).

In all, 1,744 HZ episodes were recorded in 1,553 patients (11.0%) in the overall study population. In the Cox sub-population, 362 HZ episodes were recorded in 326 patients (8.7%). Some 22% of the HZ cases in the overall study population (and 18% in the Cox subpopulation) had an AIDS-defining event prior to HZ diagnosis (Table 2). The CD4 count (cells/μL; mean ± standard deviation) at HZ diagnosis was 441 ± 259 (*n* = 1,148), while the nadir CD4 count (cells/μL; mean ± standard deviation) before HZ diagnosis was 292 ± 208 (*n* = total population). For HZ cases in the overall study population, the estimated prevalence of comorbidities at the time of HZ diagnosis was much higher than in the overall study population at the time of HIV diagnosis, with infectious diseases (excluding HZ) having the highest prevalence (42.3%).

**Table 2:** HZ episodes within the study populations

	**Total population**	**Cox**-**subpopulation**	
**Patients with an HZ episode**	**1,553(11.0%)**	**326(8.7%)**	
**Episodes**	**Total episodes**	**Total episodes**	**Initial episodes**
Number of HZ episodes	1,744	362	326
Patients with			
1 episode	1,386 (9.8%)	296 (7.9%)	Only initial
2 episodes	145 (1.0%)	24 (0.6%)	Episodes
3 episodes	20 (0.1%)	6 (0.2%)	
4 episodes	2 (0.01%)	—	
AIDS-defining event prior to HZ episode ^1^	22.1% (44 missing)	18.2% (5 missing)	18.4% (5 missing)
Mean CD4-value/μl at time of HZ-episode	441 (SD: 259) (596 missing)	434 (SD: 244) (22 missing)	423 (SD: 240) (20 missing)
Mean CD4-nadir/μl prior to HZ-episode	292 (SD: 208) (596 missing)	275 (SD: 194) (22 missing)	274 (SD: 193) (20 missing)
Infectious comorbidities at time of HZ episode	42.3%	44.8%	43.9%
Possibly IRIS associated HZ	4.14% (366 missing)	6.19% (55 missing)	6.96% (53 missing)

**Figure 2:**
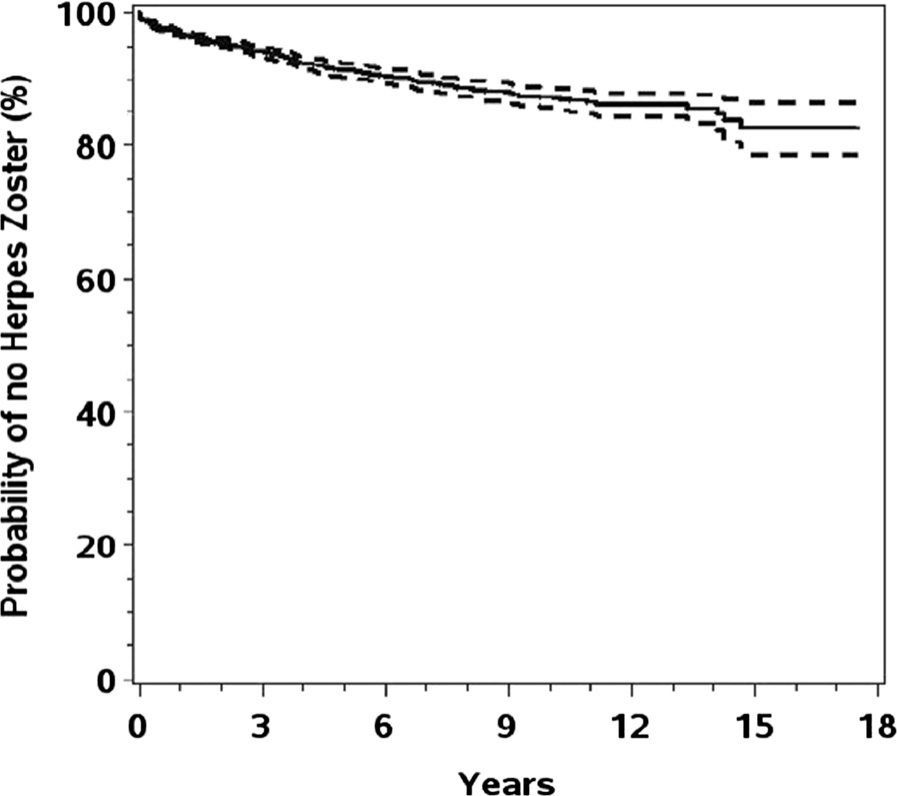
**Kaplan-Meier curve estimating cumulative Herpes Zoster probabilities.**

The overall incidences of HZ were 1.22, 1.67 and 1.61/100 person-years for the total population, all HZ cases of the Cox subpopulation and initial HZ cases of the Cox subpopulation, respectively (Table 3, *p*<0.001, Cox- vs. non-Cox subpopulations). Initial HZ episodes in the Cox subpopulation (Figure 2) showed a steady exponential decline up to 14 years. Overall, the 10-year probability of an HZ episode was 12.7% (95% CI, 11.2–14.2%). In the Cox subpopulation, 10.7% (35) of all HZ episodes occurred in the same month as HIV diagnosis, while 25.8% (84) of all initial HZ cases were observed in the first half of the year. Around 6.2% (all HZ episodes) and 7.0% (only initial HZ episodes) of cases in the Cox subpopulation were HZ episodes possibly linked to IRIS. Of the 84 HZ episodes occurring within the first 6-months after HIV diagnosis in the Cox subpopulation, 13 were linked to IRIS, 34 were unable to be classified with respect to an IRIS event.

**Table 3:** HZ incidence within the study populations

	**Total-population**	**Cox-subpopulation**	
	**All HZ cases**	**All HZ cases**	**Initial HZ cases**
Total cumulative person years	143,174	21,666	20,271
Total incidence (per 100 PY) (95% confidence intervals)	1.22 (1.16-1.28)	1.67 (1.50-1.85)	1.61 (1.44-1.79)
Age stratified incidence (95% confidence intervals)			
0-19 years	0.48 (0.17-1.06)	1.71 (0.00-9.81)	1.74 (0.00-9.98)
20-29 years	0.89 (0.77-1.03)	2.17 (1.62-2.85)	2.04 (1.50-2.72)
30-39 years	1.20 (1.11-1.29)	1.59 (1.32-1.89)	1.53 (1.27-1.84)
40-49 years	1.35 (1.24-1.46)	1.67 (1.38-2.00)	1.67 (1.37-2.01)
≥ 50 years	1.34 (1.20-1.50)	1.54 (1.19-1.96)	1.40 (1.05-1.82)

Univariate and multivariate Cox regression models were used to identify risk factors for developing an initial HZ episode. In the univariate model, hazard ratios for developing an initial HZ episode were not significant for time independent variables (sex, age, risk of transmission). Of the clinical time-dependent variables, prior exposure to ART, being on ART for 1, 2 ,3, or >4 years as opposed to no ART, having an ART-regimen containing a non-nucleoside reverse-transcriptase inhibitor (NNRTI) or a protease inhibitor (PI), or an increase in the CD4 count, significantly reduced the hazard ratio for developing an initial HZ episode, as did the retrospective mode of data documentation. Taking immune suppressive medication significantly increased the hazard ratio for developing an initial HZ episode (Table 4). Potential risk factors and confounders were also analysed. The main associated clinical risk factors for developing an initial HZ episode were: not taking an ART-regimen containing a NNRTI or a PI, and a lower CD4 count. The prospective mode of data documentation was an associated but non-clinical risk factor. Taking immune suppressive medication was no longer a risk factor after adjusting for mode of data documentation. Other significant risk factors were not influenced by adjusting the models for mode of data documentation. As in the univariate models, neither age nor sex were significant risk factors within the multivariate Cox regression models. Furthermore, a sensitivity analysis after excluding all HZ cases at HIV diagnosis (*n* = 35) by fitting the multivariate model of Table 4 did not affect the results.

**Table 4:** Univariate and multivariate analysis of risk factors associated with HZ infection

**Variable**	**Hazard ratios(95%CI)in univariate**^**1**^**models**	**P**-**value**	**Hazard ratios(95%CI)in one multivariate model**	**P-value**
Male (versus female) ^2^	0.952 (0.703, 1.290)	0.752	0.949 (0.697, 1.291)	0.739
Age (1 year change)	1.002 (0.991, 1.013)	0.728	1.002 (0.991, 1.013)	0.758
MSM (versus no MSM)	1.145 (0.905, 1.448)	0.260	(Not included)	
ART naïve, time on ART:			(Not included)	
ART-experienced	0.640 (0.455, 0.902)	0.011*		
ART-naivety uncertain	0.926 (0.640, 1.339)	0.682		
Time on ART	0.990 (0.897, 1.093)	0.841		
(1 year change)				
No ART (reference)	1.000	—		
ART naïve, time on ART classified:			(Not included)	
ART-naivety uncertain	0.940 (0.650-1.360)	0.743		
ART-experienced < 1year	0.901 (0.631-1.287)	0.567		
Time on ART 1-<2 years	0.488 (0.303-0.786)	0.003*		
Time on ART 2-<3 years	0.379 (0.219-0.655)	0.001*		
Time on ART 3-<4 years	0.469 (0.265-0.828)	0.009*		
Time on ART > 4- years	0.635 (0.364-1.108)	0.110		
No ART (reference)	1.000	—		
ART regimen, specific				
NNRTI-regimen	0.557 (0.396, 0.783)	0.001*	0.530 (0.375, 0.748)	<0.001*
PI-regimen	0.703 (0.512, 0.965)	0.029*	0.624 (0.451, 0.862)	0.004*
Regimen with other	0.717 (0.469, 1.095)	0.123	0.729 (0.469, 1.131)	0.158
Drugs/combinations				
Treatment interruption	1.153 (0.716, 1.859)	0.558	1.108 (0.686, 1.789)	0.675
No ART (reference)	1.000	—	1.000	—
CD4-rise (of 100 cells/μl)	0.939 (0.898, 0.983)	0.006*	0.918 (0.875, 0.963)	0.001*
Immunosuppressive medication (yes versus no)	1.733 (1.060, 2.832)	0.028*	1.510 (0.921, 2.477)	0.103
Retrospective documentation (yes versus no)	0.575 (0.452, 0.730)	<0.001*	0.582 (0.455, 0.745)	<0.001*

*Abbreviations*: ART: anti-retroviral therapy; NNRTI: non-nucleoside reverse-transcriptase inhibitor; PI: protease inhibitor; HZ: Herpes zoster; MSM: men who have sex with men.

^1^ For classified variables hazard ratios for each class were estimated in these “univariate” models; ^2^ two patients with missing gender were excluded from the models including male as independent variable. No other patients were excluded from the model estimations; * HR differed significantly (p<0.05) from 1 (corresponding to reference for classified variables).

## Discussion

In all, the KompNet cohort offers a very good opportunity to undertake epidemiological studies of HIV-positive patients in Germany because of the comprehensive nature of its data collection. For time-dependent risk factor analyses, we defined a Cox subpopulation. The reason for this was to avoid bias due to a large number of patients with incomplete documentation for long periods after t_0_. A further concern was that in these periods, there was a higher probability that initial HZ episodes might be missed. As the first selection criterion, a documented CD4 count within a 6-month period before and after HIV diagnosis was implemented. This was used as an indicator for the beginning of a complete course of data documentation. Furthermore, subjects with a documentation interval greater than 6 months for either CD4 count or viral load data were excluded, because documented time of HIV diagnosis might no longer be valid.

The main socio-demographic and clinical characteristics of the overall study population and the Cox subpopulation were comparable, and reflected the previously reported epidemiological characteristics of HIV-positive patients in Germany with respect to age, sex, risk of transmission of HIV, origin, and dominance of MSM and geographical origin [21,22,24]. The statistical significance of the differences between both populations were primarily because of the large population size, however the sizes of the effects regarding these epidemiological characteristics were small.

While the use of specific populations for incidence analyses and Cox regression modelling offers a reliable methodological approach, the KompNet cohort has allowed us to study the incidence of HZ infection in a much larger group of patients, with the added ability of observing patients remaining free of HZ, in contrast with previous single-centre studies [12,20].

In our study, the incidence of HZ was 1.2/100 person-years in the overall study population and 1.7/100 person-years in the Cox subpopulation. These figures are 3.6–11.3-fold higher than the 0.15–0.33/100 person-years reported for the general populations of several Western countries [1-5]. Regarding HIV-positive patients, a recent analysis by Blank et al. [20] showed an incidence of 0.9/100 person-years between 2002 and 2009, which is in contrast to earlier findings at the same HIV clinic (3.2/100 person-years between 1997 and 2001) [11]. With a high proportion of patient observations in our cohort starting after 2001 (Cox subpopulation: 75%), the results of our study are similar to the latest findings by Blank et al. [20], however we used a different study design and statistical model. It is likely that the differences between previous studies and the results of our analyses reflect a more contemporary and likely composition of current ART regimens, resulting in improved immunological conditions and thereby higher CD4 counts.

We did not observe any statistically significant differences regarding incidences stratified by age group or calendar year in either of our study populations (data not shown). The impact of older age as a risk factor for HZ in the general population may be abrogated in HIV-positive patients by other risk factors, such as the age-independent impairment of the immune system. The more or less stable incidence over time is in line with other findings [12,20] and suggests that HZ remains an important HIV-associated infection after the introduction and on-going improvement of ART options.

As expected, the mode of data documentation had some impact on the estimation of incidences. In the Cox-subpopulation, there was a small difference between the incidence of cases documented retrospectively and those documented prospectively. Further analysis using Cox regression and another stratification indicated an underestimation of the incidence in cases documented retrospectively, which was confirmed by sensitivity analyses. This may be due to a possible recall bias for long clinical histories documented retrospectively.

Kaplan Meier-analysis showed an exponential decline and therewith a more or less constant hazard for an initial HZ episode. Overall, the 10-year probability of remaining free of an initial HZ episode was 87.3%. We found that 10.7% of all HZ cases occurred at HIV diagnosis and might be the reason for HIV testing, while 25.8% of all cases were diagnosed within the first 6 months after HIV diagnosis. Of those, a relatively high proportion (26%) classifiable in terms of IRIS-related episodes had a HZ episode linked to IRIS.

We conducted Cox regression to identify risk factors for developing an initial HZ episode. In contrast to earlier studies [12], and in agreement with recent work [20], undertaking ART was one of the main protective factors in all our Cox regression models. A reason for this might be that the time of HIV diagnosis was defined as the starting point for estimating risk factors for a HZ episode, i.e. t_0_. This was much earlier than in other studies, where the starting point was often defined as the first time a subject attended a clinic, being partly clinically more progressed. Another reason could be that our analyses cover a more recent era of ART, and therefore highlight the potential benefits of modern ART regimens compared with those examined in earlier studies. In addition, our study population mainly comprised MSM, who are often highly compliant with respect to medical care and treatment, especially compared with intravenous drug users.

Constant use of immunosuppressive medication was not a risk factor in our multivariate models, probably because of their infrequent occurrence in the HIV-positive patients in our study populations. Our findings corresponded with those of Blank et al. [20], where an increase in the CD4 count was a protective factor, but with a more limited impact compared with ART. Unlike other opportunistic infections, HZ occurs at a wide range of CD4 counts in HIV-positive subjects [18]. HZ episodes in HIV-infected patients are likely to be more severe than in non-HIV-infected subjects, and can also involve recurrent episodes, more than one dermatome, systemic manifestations, and complications such as post-herpetic neuralgia [8]. In some cases, it may be the first sign of an HIV infection; while in other individuals recurrent episodes may represent progression of HIV infection [18,25]. HIV-positive subjects who develop HZ infections are known to be at high risk for progression to AIDS and death [26,27]. Therefore, an improved understanding of the incidence of and risk factors for HZ infection in HIV-positive patients has important implications for clinical practice. Our study not only clarifies the incidence in a population which reflects that seen in contemporary practice, it also indicates a positive impact of ART and increased CD4 count to avoid initial HZ episodes in HIV-positive patients.

However, our study does have some limitations. This was an observational study, and although we adjusted for available confounders including the mode of data documentation, other biases cannot be excluded. Our results regarding risk factors can only be interpreted as associations, not as causal relations. The documentation was partially retrospective, which may have contributed to a recall bias and underestimation of HZ incidence. We adjusted for this as best as possible by including a corresponding time-dependent co-variable in the Cox models. Furthermore, time-dependent CD4 values used in the Cox models were imputed using the LOCF principle between neighbouring measures or until the end of observation. This might be problematic for the time course if there are longer gaps for laboratory controls in some patients. There were 35 HZ cases at HIV diagnosis (t_0_), which may have differed from the cases during follow up. The multivariate Cox model was also fitted after excluding these 35 patients, and yielded the same result. This showed that these special HZ cases at HIV diagnosis did not bias the main results.

## Conclusions

We have shown an annual HZ incidence of 1.2/100 person-years for the overall study population, and 1.7/100 person-years for the Cox subpopulation, which is 4–11-fold higher than for non HIV-infected individuals. Incidences in our study were lower than those in previous studies, but in agreement with recently published analyses. For a large study population, this study showed ART as the most important protective factor for HZ episodes.

## Competing interests

KJ, BH, CK, ASR, SE declare that they have no competing interests. AG is an employee of GlaxoSmithKline Biologicals. SD has received congress sponsorship, and has had consultancy and advisory relationships with Abbott, Boehringer Ingelheim, Bristol-Myers-Squibb, and Gilead. AP has received congress sponsorship, and has had consultancy and advisory relationships, and received research funding from Abbott, Boehringer Ingelheim, Bristol-Myers-Squibb, Essex, Gilead, GlaxoSmithKline Biologicals, ViiV Healthcare, MSD Sharp & Dohme, Pfizer, Roche, Janssen-Cilag, Sanofi Pasteur, and Novartis. NHB has received congress sponsorship, and has had consultancy and advisory relationships, while also receiving research funding from Abbott, Boehringer Ingelheim, Bristol-Myers-Squibb, Essex, Gilead, GlaxoSmithKline Biologicals, ViiV Healthcare, MSD Sharp & Dohme, Pfizer, Roche, Janssen-Cilag, and Sanofi Pasteur.

## Authors’ contributions

KJ, BH, CM, AG and NHB conceived the study and participated in its design. KJ and BH conducted the statistical analyses and drafted the manuscript. SE, SD, AP and ASR participated in the study design and coordination. All authors read and approved the final version of this manuscript.

## Pre-publication history

The pre-publication history for this paper can be accessed here:

http://www.biomedcentral.com/1471-2334/13/372/prepub
